# Therapeutic potential of sulforaphane in liver diseases: a review

**DOI:** 10.3389/fphar.2023.1256029

**Published:** 2023-08-29

**Authors:** Liang Yan, Yachun Yan

**Affiliations:** Department of Pharmacy, The First Affiliated Hospital of Zhengzhou University, Zhengzhou, China

**Keywords:** sulforaphane, Nrf2, metabolic liver disease, antioxidant, autophagy

## Abstract

The burden of liver diseases such as metabolic-associated fatty liver diseases and hepatocellular carcinoma has increased rapidly worldwide over the past decades. However, pharmacological therapies for these liver diseases are insufficient. Sulforaphane (SFN), an isothiocyanate that is mainly found in cruciferous vegetables, has been found to have a broad spectrum of activities like antioxidation, anti-inflammation, anti-diabetic, and anticancer effects. Recently, a growing number of studies have reported that SFN could significantly ameliorate hepatic steatosis and prevent the development of fatty liver, improve insulin sensitivity, attenuate oxidative damage and liver injury, induce apoptosis, and inhibit the proliferation of hepatoma cells through multiple signaling pathways. Moreover, many clinical studies have demonstrated that SFN is harmless to the human body and well-tolerated by individuals. This emerging evidence suggests SFN to be a promising drug candidate in the treatment of liver diseases. Nevertheless, limitations exist in the development of SFN as a hepatoprotective drug due to its special properties, including instability, water insolubility, and high inter-individual variation of bioavailability when used from broccoli sprout extracts. Herein, we comprehensively review the recent progress of SFN in the treatment of common liver diseases and the underlying mechanisms, with the aim to provide a better understanding of the therapeutic potential of SFN in liver diseases.

## 1 Introduction

Over the past decades, liver diseases have become one of the major diseases threatening human health globally, with the burden of liver diseases increasing rapidly (Pimpin et al., 2018; Sarin et al., 2020). Although plenty of studies have been published focusing on the pathogenesis and regulatory mechanisms of different kinds of liver diseases, effective strategies for the prevention and treatment of liver diseases are still insufficient. Recently, a number of natural products have been shown to be effective in the treatment of liver diseases, such as fatty liver disease and drug-induced liver injury, which provides a new direction for the development of drugs to treat liver diseases (Chen et al., 2017; Tarantino et al., 2021; Sun et al., 2022). Sulforaphane (SFN) is an aliphatic isothiocyanate derived from the hydrolysis of glucoraphanin catalyzed by myrosinase in plants, which is abundant in cruciferous vegetables like broccoli, cabbage, and brussels sprouts (Kaiser et al., 2021). Since it was discovered, the beneficial effects of SFN on health have been widely reported, such as antioxidative, anti-inflammatory, antidiabetic, anticancer, neuroprotective, and cardiovascular protective activities, along with having low toxicity to the human body (Greaney et al., 2016; Feng et al., 2017; Patel et al., 2018; Houghton, 2019; Calabrese and Kozumbo, 2021). In recent years, clinical trials have been performed to assess the therapeutic efficacy of SFN in the treatment of several diseases, such as different types of cancer, neuro-degenerative diseases, autism spectrum disorder, and non-alcoholic fatty liver disease (NAFLD) (Singh et al., 2014; Schepici et al., 2020; Mangla et al., 2021). The effects of SFN on chronic and acute liver diseases were investigated more than a decade by studies using *in vitro* or *in vivo* models (Kim et al., 2006; Zhao et al., 2010; Xu et al., 2019). Previous studies have indicated that the antioxidative activity and biological functions of SFN are partially dependent on the activation of nuclear factor-erythroid 2-related factor 2 (Nrf2), which is a master regulator of cellular homeostasis that is ubiquitously expressed in many organs (Houghton et al., 2016; Cuadrado et al., 2018; Zhou et al., 2022). The beneficial effects of SFN on lipid metabolism and NAFLD through the activation of Nrf2 have been reviewed previously (Xu et al., 2019; Du et al., 2021). However, with a growing number of studies focusing on the role of SFN in liver diseases, increasing pathways and targets that are mediated by SFN have been discovered. In this review, the efficacy and related mechanisms of SFN in the treatment of liver diseases, including fatty liver disease, hepatic insulin resistance, xenobiotic-induced liver injury, hepatic ischemia-reperfusion injury, and hepatocellular carcinoma (HCC), were summarized. Additionally, strategies for solving problems of SFN like instability and insolubility, as well as a selection of formulation and dose schedules in clinical trials, were discussed, with the aim to provide insights into the future studies of SFN in the treatment of liver diseases.

## 2 The bioavailability, pharmacokinetics, and safety of SFN

SFN is a sulfur-containing compound that is insoluble in water, but glucoraphanin, its precursor and storage form in plants, is water-soluble and is used more frequently in clinical research either in its pure form or in the form of broccoli seed and sprout extracts. On average, the bioavailability of pure SFN is about seven times that of glucoraphanin (Shapiro et al., 2006; Egner et al., 2011; Fahey et al., 2012). However, the source materials that were used in clinical studies showed significant influence on the bioavailability of SFN in human body. For instance, the peak plasma concentration of SFN in subjects who received fresh broccoli sprouts was 7-fold higher than that of broccoli supplements, and the urinary excretion was 5-fold higher, which indicated a much higher bioavailability of SFN in humans when administered from a whole food source (Clarke et al., 2011a). The reason was thought to be due to the presence of myrosinase in fresh broccoli sprouts (Fahey et al., 2015). Thus, a dietary supplement containing myrosinase could dramatically increase the bioavailability of SFN in human subjects receiving cooked broccoli (Okunade et al., 2018). Moreover, different cooking methods were also reported to affect the bioavailability of SFN (Orlando et al., 2022). The bioavailability of SFN was found to be affected by gastric acidity when broccoli seeds were co-delivered, which might be associated with altered activity of myrosinase as it could be improved by the use of omeprazole or enteric coating of myrosinase (Fahey et al., 2019). Additionally, the bioavailability of SFN was also reported to be influenced by gut microflora because of its ability to secrete myrosinase and convert glucoraphanin to SFN (Tian et al., 2018b).

SFN is absorbed and metabolized quickly in the human body after ingestion. The plasma concentration of SFN peaks within 3 h and is cleared from the body in 24 h, with a half-life of about 2 h, which might be slightly affected by the formulation used in different studies (Al Janobi et al., 2006; Egner et al., 2008; Atwell et al., 2015). Once entering systemic circulation, SFN is distributed to several organs, like the small intestine, prostate, kidney, and lung (Clarke et al., 2011b; Livingstone et al., 2022). In the human body, SFN is primarily metabolized via the mercapturic acid pathway. Under the catalysis of glutathione transferases, SFN is conjugated with glutathione (GSH) to form SFN-GSH, then metabolized to SFN-cysteinylglycine, SFN-cysteine, and SFN-N-acetylcysteine under the catalyzation of γ-glutamyltranspeptidase, cysteinylglycinase, and N-acetyltransferase, respectively (Kolm et al., 1995; Egner et al., 2008). Subsequently, these metabolites are excreted in urine (Conaway et al., 2000; Ye et al., 2002). Some SFN could be reversibly converted to erucin by oxidation *in vivo*, which also demonstrated similar biological activities (Clarke et al., 2011a).

Although SFN showed anticancer activity and cytotoxicity to cancer cells, it is relatively safe for normal cells. For instance, SFN inhibited the viability of about 70% of HepG2 cells after exposure for 48 h at 20 μM, but no significant cytotoxicity was observed in cultured human hepatocytes when exposed to 50 μM of SFN for 48 h (Park et al., 2007; Gross-Steinmeyer et al., 2010). Moreover, the safety of SFN was also confirmed in clinical studies as no severe adverse effects were observed in participants who were administrated SFN either for a short period or for several weeks (Kensler et al., 2005; Shapiro et al., 2006; Alumkal et al., 2015). Occasionally, a few mild adverse effects of SFN were observed in clinical studies, like grade 2 constipation, nausea, headache, and bloating (Alumkal et al., 2015; Tahata et al., 2018; Zhang et al., 2020). But SFN treatment did not increase the risk of side effects in patients (Ghazizadeh-Hashemi et al., 2021). In a 15-week randomized parallel double-blind placebo-controlled clinical trial in children with autism spectrum disorder, SFN was found to be associated with insomnia, irritability, and intolerance of taste and smell, but no severe adverse effects were observed and serum chemistry profile, urinalysis, and complete blood count of patients were normal (Zimmerman et al., 2021). Collectively, SFN is regarded as safe to the human body when used within limited doses.

## 3 The efficacy of SFN in the treatment of liver diseases

SFN has multiple effects on the liver. Initially, it was shown to induce detoxifying and antioxidant enzymes in the liver, which consequently mediates the detoxification and clearance of carcinogens and reactive oxygen species to elevate cell defense (Hu et al., 2004; Hu et al., 2006). As well as chemopreventive and anti-cancer activities, many studies have reported the protective role of SFN in oxidative stress-induced liver injury and hepatic inflammation caused by drugs, alcohols, toxins, and so forth. In recent years, emerging studies have found that SFN affects the metabolism of glucose and lipids in the liver, and could improve insulin resistance and reduce hepatic lipid accumulation. In this part, the efficacy of SFN in the treatment of several liver diseases, including fatty liver disease, xenobiotic-induced liver injury, hepatic insulin resistance, hepatic ischemia-reperfusion injury, and HCC, as well as related mechanisms, will be discussed. Research data from preclinical and clinical studies about the effects of SFN on liver diseases were collected from PubMed, Web of Science, and the scientific Databases of Science Direct. Keywords included sulforaphane, steatosis, fatty liver, insulin resistance, hepatotoxicity, liver injury, ischemia reperfusion, and hepatocellular carcinoma and were used to retrieve literature published before 31 January 2023.

### 3.1 SFN and fatty liver disease

Fatty liver disease (FLD) is a common liver disease around the world with a broad spectrum from simple steatosis to steatohepatitis, fibrosis, and cirrhosis, and is characterized by excessive lipid accumulation in the liver (Louvet and Mathurin, 2015; Cotter and Rinella, 2020). Patients with hepatic steatosis are usually diagnosed with alcoholic fatty liver disease (AFLD) or nonalcoholic fatty liver disease (NAFLD) according to the consumption of alcohol or not. Heterogeneity in the pathogenesis of FLD has been recognized by clinicians and scientists (Eslam et al., 2020). However, this disease has been found to be accompanied by several physiological characters like inflammation, endoplasmic reticulum (ER) stress, oxidative stress, and mitochondrial dysfunction (Bessone et al., 2019; Arroyave-Ospina et al., 2021). The usage of natural compounds from herbs in the treatment of liver diseases has been applied for a long time. Recently, the beneficial effects of phytochemical active compounds like SFN, silybin, and curcumin on FLD have been proven by both experimental and clinical studies, mostly due to the antioxidative and anti-inflammation activities (Bagherniya et al., 2018; Cheng et al., 2019; Du et al., 2021).

**FIGURE 1:**
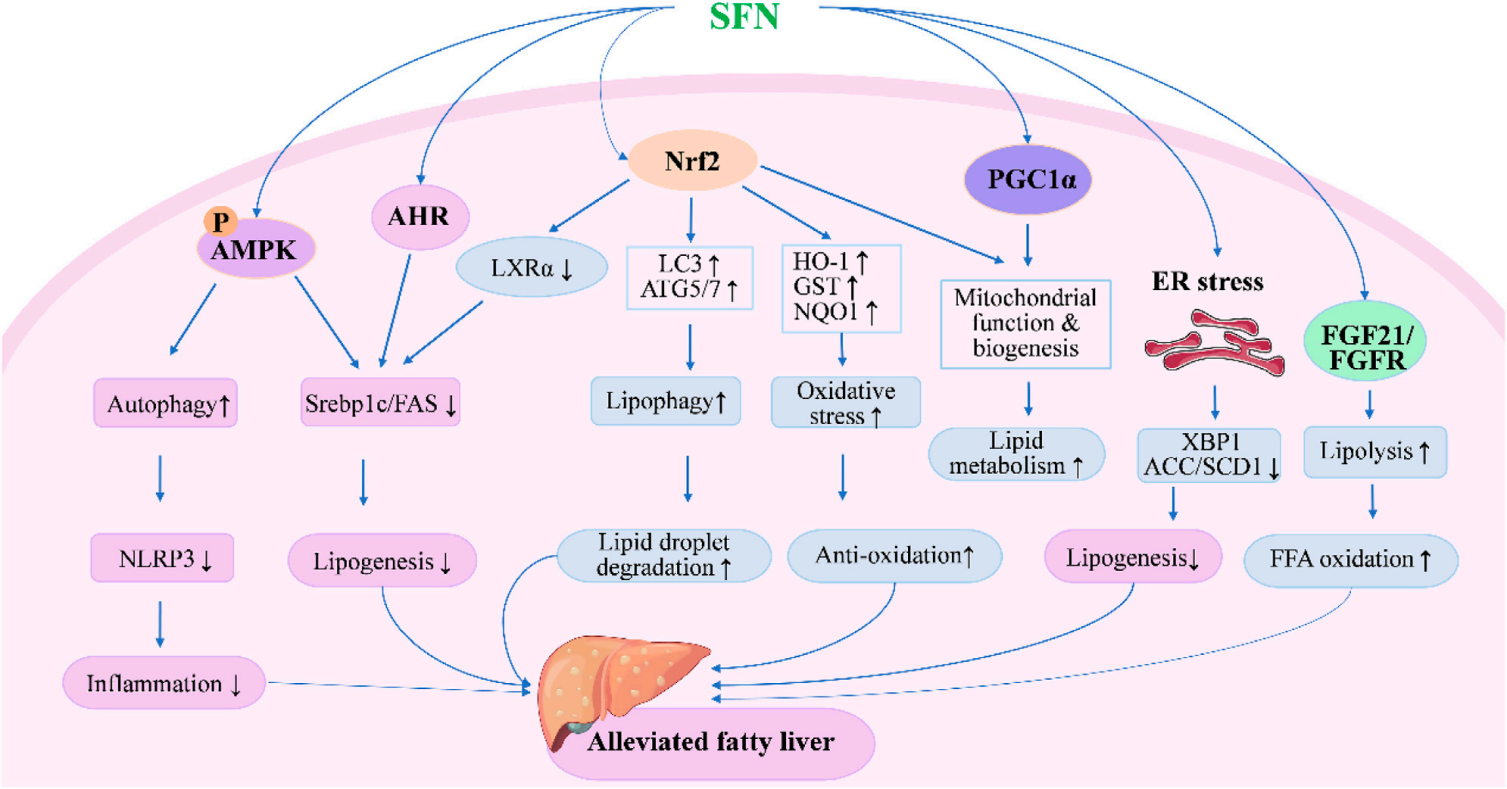
Molecular mechanisms under the efficacy of SFN in the treatment of fatty liver disease. SFN could alleviate fatty liver either through Nrf2-dependent or independent pathways. Upon activation of Nrf2, SFN inhibits lipogenesis and oxidative stress while enhancing lipid droplet degradation through modulating the expression of genes involved in lipid synthesis, metabolism, and oxidation. Moreover, SFN modulates autophagy, lipolysis, mitochondrial function, and ER stress to alleviate fatty liver through AMPK-, AHR-, PGC1α-, and FGF21-mediated pathways.

SFN was first reported to activate Nrf2 and prevent liver X receptor-α (LXRα) agonist-induced hepatic lipogenesis and steatosis by activating farnesoid X receptor (FXR) and inducting small heterodimer partner in mice in 2011 (Kay et al., 2011). The following year, a study reported that coadministration of SFN with a methionine- and choline-deficient diet significantly decreased inflammatory cell infiltration and suppressed fibrosis and oxidative stress in the livers of mice with steatohepatitis (Okada et al., 2012). More recently, SFN was found to enhance lipid droplet degradation and ameliorate steatosis in hepatocytes and rat liver through Nrf2-mediated lipophagy (Lei et al., 2022). The efficacy of SFN in preventing alcohol-induced hepatic steatosis has also been reported: the intake of SFN showed protective effects against ethanol-induced liver injury and alleviated steatosis in mice, which were mainly associated with the upregulation of antioxidant capacity and suppression of ER stress through Nrf2 pathway (Zhou et al., 2014; Lei et al., 2018; Wang and Zhou, 2020). Apart from activation of Nrf2, SFN was also shown to prevent high-fat diet (HFD)-induced NAFLD in mice by inhibiting NOD-like receptor family pyrin domain containing 3 (NLRP3) inflammasome in liver through AMP-activated protein kinase (AMPK)-autophagy axis when administered with a daily dose of 30 mg/kg for 9 weeks along with HFD (Yang et al., 2016). In recent years, the number of new mechanisms and molecular targets of SFN in the treatment of fatty liver reported by experimental studies has increased rapidly (Figure 1), such as inhibition of lipogenic enzymes via ER stress-dependent decrease of X-box binding protein 1 (XBP1) expression and ER stress-independent blocking of sterol regulatory element binding protein-1c (SREBP1c) pathways (Tian et al., 2018a), alleviated ER stress through the upregulation of AMPK and peroxisome proliferators-activated receptor α (PPARα) (Mansour et al., 2022), enhanced mitochondrial function via Nrf2 activation or promotion of mitochondrial biogenesis by peroxisome proliferator-activated receptor alpha co-activator pathway (Lei et al., 2019), and regulation of FXR-mediated bile acid metabolism and LXRα-mediated fatty acid synthesis pathways (Ma et al., 2022). SFN was also found to alleviate HFD-induced lipid deposition and suppress apoptosis by regulating the AMPK/SREBP1c/FAS signaling pathway both *in vitro* and *in vivo* (Li et al., 2021). In addition, fibroblast growth factor 21 (FGF21), which is expected to be a promising therapeutic target for the treatment of hepatic steatosis due to its beneficial effects on lipid metabolism in liver (Fisher and Maratos-Flier, 2016), was newly reported to be a target of SFN (Tian et al., 2021b). A continuous administration of SFN for 6 weeks significantly increased hepatic expression of FGF21 and facilitated fatty acid metabolism through preserving FGF receptor 1 protein and preventing the phosphorylation of p38MAPK in HFD-induced NAFLD mice (Wu et al., 2022). In addition, gut microbiota and gut microbiome-derived metabolite, indole-3-acetic acid, were also reported to contribute to the efficacy of SFN in improving HFD-induced hepatic steatosis along with Aryl hydrocarbon receptor (AHR)/SREBP1c pathway-mediated lipid metabolism (Xu et al., 2021). Together, these experimental studies suggest the great potential of SFN in the treatment of FLD.

Although clinical studies for SFN have been performed for many years, there has been little focus on the efficacy in treatment of fatty liver disease. Until now, only one published clinical study has reported the effect of SFN-containing broccoli sprout extract on fatty liver in humans. In that randomized, placebo-controlled, and double-blind trial, daily dietary supplementation with broccoli sprout extract containing glucoraphanin, a precursor of SFN, showed a positive effect on improving liver function in male subjects with fatty liver. The levels of serum biomarkers of liver function, including alanine aminotransferases (ALT), γ-glutamyl transpeptidase (γ-GT), alkali phosphatase (ALP), and urinary level of 8-OHdG, an oxidative stress marker, were significantly decreased in subjects after a daily supplementation of broccoli sprout extract containing 30 mg of glucoraphanin for 2 months (Kikuchi et al., 2015). Although only a small group of subjects were enrolled and broccoli sprout extract was used instead of SFN, the findings in this study are still valuable. A clinical trial focusing on the efficacy of dietary supplementation of SFN in treatment of NAFLD, obesity, and metabolic syndrome is still ongoing (Retrieved from https://clinicaltrials.gov/ct2/show/NCT04364360). Obviously, more clinical studies about the efficacy of SFN in the treatment of FLD are meaningful and anticipated.

### 3.2 SFN and hepatic insulin resistance

Under normal conditions, insulin regulates the homeostasis of glucose and lipid metabolism in liver by direct or indirect signal pathways (Titchenell et al., 2017). But hepatic insulin resistance occurs in some pathologic states, such as obesity and type 2 diabetes, which leads to an increase of hepatic glucose production and lipid synthesis (Santoleri and Titchenell, 2019). Thus, hepatic insulin resistance is closely linked to NAFLD. Several therapies have been applied in clinical practice to treat hepatic insulin resistance, like weight loss with diet or bariatric surgery, aerobic exercise, and pharmacological strategies (Samuel and Shulman, 2018). Antioxidants are considered as a new therapy for the treatment of insulin resistance and diabetes because of their association with oxidative stress. In a parallel, randomized, double-blind, and placebo-controlled clinical study, broccoli sprout powder containing high amounts of SFN was found to decrease serum concentration of insulin and a homeostasis model assessment of insulin resistance indices in patients with type 2 diabetes (Bahadoran et al., 2012). This suggests the therapeutic potential of SFN in the treatment of insulin resistance.

In high-fructose-fed rats, treatment of SFN showed comparable improvements of insulin resistance and hepatoprotective effect when compared to the standard insulin sensitizer pioglitazone (Shawky et al., 2019). Experimentally, both pre- and post-treatment with SFN significantly elevated insulin sensitivity and hepatic glycogen concentration in streptozotocin-induced diabetes in rats but did not impact antioxidant response in the liver (de Souza et al., 2012; de Souza et al., 2016). Long-term coadministration of glucoraphanin with HFD in mice showed benefits for ameliorating insulin resistance and hepatic steatosis in a Nrf2-dependent manner, and these benefits might be attributed to the inhibition of metabolic endotoxemia-related chronic inflammation and oxidative stress by glucoraphanin (Nagata et al., 2017). Except for alleviating hepatic steatosis, SFN showed antidiabetic effects and ameliorated insulin resistance either by enhancing antioxidant capacity and improving FGF21 resistance in HFD and streptozotocin-induced type 2 diabetes or through activating AMPK/Nrf2-mediated antioxidative effect via inactivation of glutathione peroxidase 4 in HFD-induced insulin resistance in mice (Tian et al., 2021a; Zhang et al., 2022). Through the activation of Nrf2, SFN inhibited hepatic glucose production and decreased the expression of key enzymes in gluconeogenesis in HFD-induced diabetic rats, and reversed the gene expression profiles of the hepatic disease signature of diabetes. Moreover, clinical data suggested that the antidiabetic effect of SFN was found to be most effective in obese patients with dysregulated type 2 diabetes without causing severe adverse effects (Axelsson et al., 2017).

**FIGURE 2:**
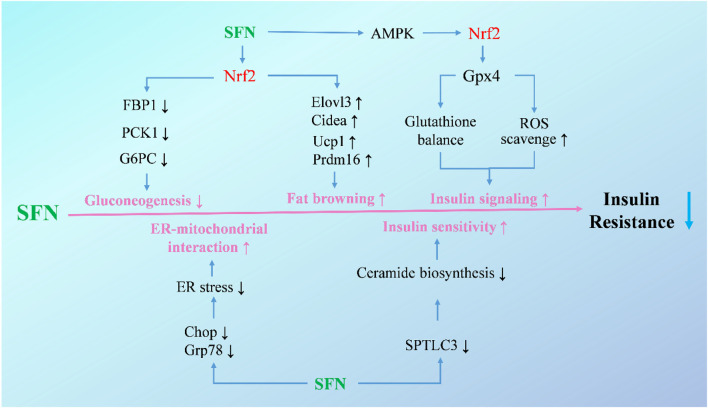
Molecular mechanisms under the efficacy of SFN in the treatment of hepatic insulin resistance. Upon activation of Nrf2, SFN inhibits gluconeogenetic enzymes and decreases gluconeogenesis to attenuate exaggerated glucose production and glucose intolerance, increases brown fat-selective genes, and promotes adipose tissue browning to mitigate obesity and insulin resistance. Additionally, SFN increases insulin signaling in hepatocytes and ameliorates HFD-induced insulin resistance through activating the AMPK-Nrf2-Gpx4 pathway. SFN also alleviates ER stress to enhance ER-mitochondrial interaction and blocks ceramide biosynthesis to improve insulin sensitivity.

Additionally, new targets of SFN in the treatment of hepatic insulin resistance have been identified by researchers in recent years (Figure 2). The mitochondria-associated ER membranes are considered as hubs for hepatic metabolism and are tightly related with gluconeogenesis in hepatocytes. Disruption of mitochondria-ER interactions occurs in the early stage of hepatic insulin resistance, and targeting mitochondria-ER interactions has been proven to be effective in improving insulin resistance in diabetic mice (Tubbs et al., 2014; Beaulant et al., 2022). In a recent study, SFN showed a significant effect on restoring mitochondria-ER interactions along with alleviating hepatic insulin resistance and glucose intolerance both *in vitro* and *in vivo*, with a disruption of mitochondria-ER interactions in hepatocytes or mice induced by HFD and high-sucrose diet counteracted by coadministration of SFN (Tubbs et al., 2018). Apart from that, ceramides, which have been shown to correlate with lipid-induced hepatic insulin resistance, were also reported to be a target of SFN (Petersen and Shulman, 2017; Chaurasia et al., 2019; Teng et al., 2019). In insulin-resistant HepG2 cells, SFN treatment counteracted palmitic acid-induced increase of ceramides, and alleviated insulin resistance through blocking ceramide biosynthesis by reducing the expression of serine palmitoyltransferase 3. Moreover, long-term treatment of SFN completely normalized hepatic ceramide levels and improved insulin sensitivity in HFD-induced mice (Teng et al., 2019).

### 3.3 SFN and xenobiotic-induced liver injury

**FIGURE 3:**
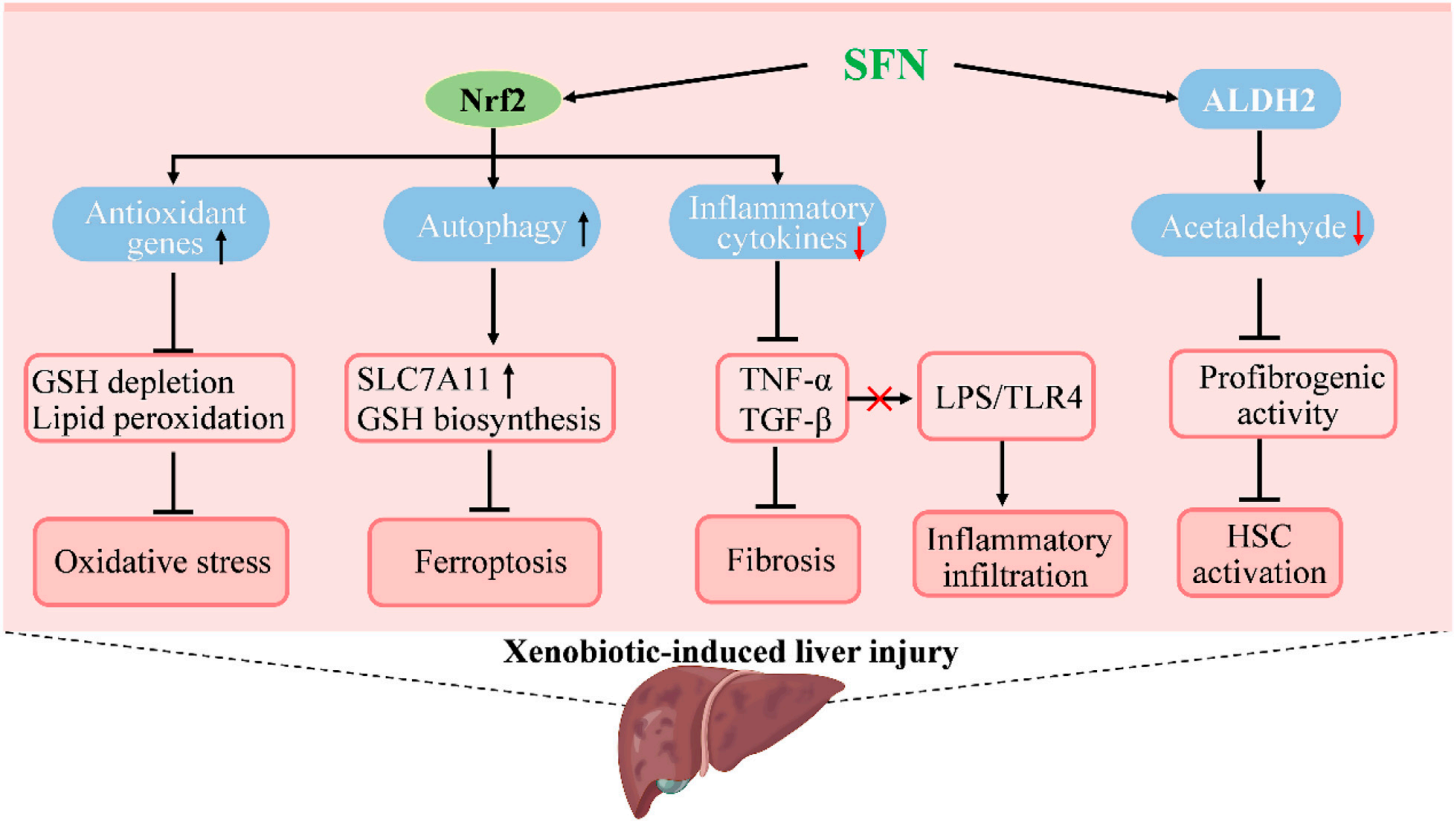
Molecular mechanisms under the efficacy of SFN in the treatment of xenobiotic-induced liver injury. SFN activates Nrf2 to increase the expression of genes in antioxidation and autophagy to suppress oxidative stress and ferroptosis, which prevents or attenuates xenobiotic-induced liver injury. The activation of Nrf2 by SFN also decreases inflammatory cytokines to inhibit inflammatory infiltration and hepatic fibrosis that occurs in liver injury. Moreover, SFN induces the activity of acetaldehyde-metabolizing enzyme ALDH2 to promote acetaldehyde metabolism and suppresses acetaldehyde-induced proliferation and profibrogenic activity of hepatic stellate cells to ameliorate alcoholic liver injury.

Liver injury induced by xenobiotics, such as drugs, endocrine-disrupting chemicals, pollutants, and herbs, is an important part of liver diseases that could lead to liver failure or even death (Fontana et al., 2014; Andrade et al., 2019). Prevention and treatment of xenobiotic-induced liver injury are rather challenging due to the difficulties in diagnosis and incomplete understanding of pathogenesis, especially of idiosyncratic liver injuries (Fontana, 2014; Hassan and Fontana, 2019). However, several studies have reported the protective role and possible mechanisms of SFN in xenobiotic-induced liver injury by using *in vivo* models (Figure 3). In acetaminophen-induced acute liver damage in mice, pretreatment with SFN showed a protective effect against severe liver injury and oxidative stress by inhibition of reactive oxygen species (ROS) formation and induction of heme oxygenase-1 (HO-1) expression (Noh et al., 2015). The hepatic expression of genes related to detoxification and GSH synthesis were induced by broccoli sprout extract treatment, which might contribute to the liver-protective effect of SFN in acetaminophen-induced injury (Yoshida et al., 2015). The antioxidant activity of SFN and its effect on GSH synthesis are similar to that of N-acetyl cysteine, an accomplished antioxidant that has been widely used clinically in the treatment of acetaminophen-induced injury (Ntamo et al., 2021; Schwalfenberg, 2021). The hepatic protective effect of SFN was also reported in cisplatin-induced liver injury. Pretreatment with SFN in rats could prevent hepatic damage induced by cisplatin and the decrease of antioxidant enzyme activity as well as mitochondrial alterations in oxygen consumption were also attenuated (Gaona-Gaona et al., 2011). Further, coadministration of SFN showed a protective effect in sodium valproate-induced liver injury in rats, and reduced the content of malondialdehyde (MDA) and tumor necrosis factor α (TNFα), while the concentration of GSH and HO-1 were increased (Nazmy et al., 2017). Olanzapine is a widely used antipsychotic drug, but side effects such as weight gain, dyslipidemia, and liver injury are common. In a HFD plus olanzapine-induced chronic liver injury model in mice, SFN was found to effectively prevent the exacerbated liver damage caused by the interaction of olanzapine and HFD; moreover, fat accumulation and inflammation were significantly decreased along with 4-HNE, a biomarker of oxidative/nitrosative stress (Isaacson et al., 2020).

Apart from drug-induced liver injury, the role of SFN in acute liver injuries induced by other substances has been studied, like carbon tetrachloride (CCl_4_), D-galactose, lipopolysaccharide (LPS), nanoparticles, and environmental toxicants. Oral administration of SFN after CCl_4_ significantly decreased serum ALT in mice and reduced necrotic zones and lipid peroxidation in the liver (Baek et al., 2008). Liver fibrosis caused by ethanol/CCl_4_ in mice was also ameliorated by SFN treatment, possibly through Nrf2-mediated antioxidation and enhanced acetaldehyde metabolism (Ishida et al., 2021). Recently, SFN was found to reduce ferroptosis in acute liver injury in an Nrf2-dependent manner. Upon activating Nrf2, SFN induced autophagy and upregulated the expression of solute carrier family 7 member 11 and promoted its membrane transfer, which finally suppressed ferroptosis to alleviate acute liver injury both *in vitro* and *in vivo* (Liu et al., 2023). Because of its antioxidant and anti-inflammatory capacity, SFN showed benefits not only in alleviating liver damage and reducing mortality in D-galactose/LPS-induced fulminant hepatic failure, but also in prevention of hepatic fibrosis and injury caused by D-galactose in rats (Sayed et al., 2014; Saleh et al., 2019). However, although anti-inflammatory effects were obvious in the liver, no improvement of LPS-induced sickness behavior in mice was observed by a short pretreatment with SFN (Townsend and Johnson, 2017). In the study of cadmium selenide quantum dots-induced hepatotoxicity, treatment with SFN significantly reduced cell death in human hepatocytes and decreased liver damage in mice through the induction of Nrf2 pathway and autophagy (Wang et al., 2015). In addition, SFN was found to prevent arsenic-induced hepatotoxicity in mice by PI3K/Akt mediated Nrf2 signaling pathway, which suggests a protective effect of SFN on toxicant-induced liver injury (Thangapandiyan et al., 2019). Though the efficacy of SFN in preventing liver injury or in therapy of xenobiotic-induced liver injuries has been demonstrated by various experimental studies, clinical data from patients are lacking. In view of the therapeutic potential, evaluating the effect of SFN on xenobiotic-induced liver injuries in patients in the future is meaningful.

### 3.4 SFN and hepatic ischemia-reperfusion injury

Hepatic ischemia-reperfusion injury (IRI) is a major complication of hemorrhagic shock and transplantation and is the most difficult problem in liver transplantation, often leading to liver dysfunction or failure of transplantation. Hepatic IRI is considered as a local proinflammatory response mediated by the innate immune system (Zhai et al., 2013). Although the mechanisms of hepatic IRI are largely unknown, several targets have been revealed to be associated with its pathogenesis by preclinical studies such as proliferators-activated receptor γ, family with sequence similarity 3A, and some noncoding RNAs (Yang et al., 2018). Targeting Nrf2 through SFN was found to be effective in handling hepatic IRI due to its antioxidative activity. This suggests that SFN could be useful in the treatment of hepatic IRI, since clinical trials have reported that antioxidants like N-acetyl cysteine could improve liver function of patients with liver transplantation by inhibiting oxidative stress and inflammation (Ntamo et al., 2022). In hepatic IRI rats, pretreatment with SFN could alleviate liver injury by inhibiting oxidative stress and improving antioxidative activity as well as mitochondrial function in liver tissues, which was accompanied with the activation of Nrf2 pathway (Zhao et al., 2010; Chi et al., 2015; Oguz et al., 2015). Upregulation of carbonyl reductase 1, an enzyme that protects cells against oxidative stress and cell death by inhibiting the formation of lipid peroxides, was observed in SFN-pretreated hepatic IRI mice in an Nrf2-dependent manner (Kwon et al., 2019). Apart from antioxidation, an anti-inflammatory effect was also involved in the protection of hepatic IRI by SFN. The upregulation of inflammatory cytokines in hepatic IRI rats, like TNFα, Interleukin 6, and Monocyte chemoattractant protein-1, were inhibited by SFN treatment which was dependent on the activation of Nrf2 (Chen et al., 2021). More recently, immunomodulatory effects of SFN in alleviating hepatic IRI were further identified in a murine model. In male C57BL/6 mice with hemorrhagic shock followed by resuscitation, SFN treatment reduced inflammatory cytokine secretion of Kupffer cells, decreased the infiltration of neutrophils in the liver, and alleviated liver damage through the Nrf2-mediated pathway (Liang et al., 2022). Together, these results indicate that antioxidative and anti-inflammatory effects are indispensable for SFN in the treatment of hepatic IRI.

### 3.5 SFN and hepatocellular carcinoma

The antineoplastic activity of SFN has been widely studied since its discovery in the 1990s, and multiple molecular pathways have been revealed, including HCC (Kaiser et al., 2021). SFN exhibited antiproliferative activity in HepG2 cells by inducing apoptosis through the activation of caspase-3 and upregulation of Bcl-2-associated X protein, as well as downregulation of Bcl-2 and Bcl-XL expression (Park et al., 2007). In another study, SFN-mediated apoptosis in Huh7 cells was found to be associated with the inhibition of 6-phosphofructo-2-kinase/fructose-2,6-biphosphatase 4 expression and HIF-1α pathway, and the inhibition was even higher under hypoxic conditions (Jeon et al., 2011). Moreover, SFN suppressed angiogenesis and tumor growth by inhibiting STAT3/HIF-1α/VEGF pathway in HepG2 cells and tumor tissues as the expression of HIF-1α, STAT3, and VEGF were decreased (Liu et al., 2017). In addition, the biomarkers of ER stress, like C/EBP homologous protein and XBP-1, were upregulated in apoptotic HepG2 cells induced by SFN treatment, suggesting the involvement of ER stress in SFN-induced apoptosis (Zou et al., 2017). Apart from apoptosis, SFN treatment inhibited the formation of fibroblast-like mesenchymal cells and expression of vimentin in HepG2 cells, while it increased the expression of E-cadherin, which indicates that SFN suppresses epithelial-mesenchymal transition in HCC (Wu et al., 2016). In Hep3B cells, SFN not only decreased cell viability but also inhibited telomerase activity via reducing the expression of telomerase reverse transcriptase in a ROS-dependent manner, suggesting a novel mechanism of the antineoplastic activity of SFN (Moon et al., 2010). Recently, active metabolites of SFN, such as SFN-GSH, SFN-cysteine, and SFN-*N*-acetylcysteine, were found to have similar chemopreventive activities to SFN in HepG2 cells (Liu et al., 2018). Furthermore, SFN was found to be a potent sensitizer for tumor necrosis factor-related apoptosis-inducing ligand-induced apoptosis in hepatoma cells through the generation of ROS and subsequent upregulation of death receptor 5 (Kim et al., 2006). Together, these *in vitro* findings suggest an effective role of SFN in the treatment of HCC.

However, *in vivo* studies on the chemopreventive activity of SFN in HCC are very limited. In a HepG2 cell-derived xenograft tumor model in Balb/c athymic nude mice, treatment with SFN for 13 days significantly inhibited tumor growth and reduced the volume of tumors (Wu et al., 2016). But in another *in vivo* study, an SFN-containing diet did not prevent the hepatic tumorigenesis induced by the diethylnitrosamine in C57BL/6J mice (Chen et al., 2016). In this study, broccoli powder was used instead of SFN and administered along with a western diet during the study period. Although SFN content was 4 mmol/kg broccoli powder in the present study, the source, dose, and route of administration of SFN may affect its final efficacy as it is metabolized quickly in the body with a relatively short half-life (Yagishita et al., 2019).

## 4 Future perspectives

SFN has become a promising phytochemical in the treatment of several diseases in the last few years, including various cancers, autism spectrum disorder, schizophrenia, obesity, and fatty liver disease. Although few clinical trials have focused on the efficacy of SFN in the treatment of liver diseases, previous findings from preclinical studies are exciting, especially in fatty liver disease. Thus, advanced research and clinical trials that focus on the efficacy of SFN in the treatment of liver diseases are necessary in the future. Nevertheless, there are some problems that need to be solved by future research.

Firstly, the development of SFN is limited by its water-insoluble and unstable properties. SFN is sensitive to temperature and easy to degrade in aqueous and protic solvents and polar aprotic environments (Franklin et al., 2014). Developing strategies to improve the stability of SFN in formulation is an important direction for future research. Novel drug delivery systems are expected to be suitable candidates in future studies. Previously, a commercially available product of stabilized SFN was developed, named Prostaphane^®^, but its storage condition is limited to between 4°C–8°C. Besides, novel delivery systems have been reported to successfully increase the stability and solubility of SFN in recent years, such as coated microparticles, microencapsulation, and nanoparticles, and these strategies for improving the stabilization of SFN have been discussed in a recent review (Yuanfeng et al., 2021). Nevertheless, it is worth noting that most of these formulations remain to be tested on the human body to evaluate their safety, as some of these materials may cause unwanted effects. For instance, a high incidence of mild stomach upset has been observed in a clinical trial of α-cyclodextrin inclusion of SFN (Fahey et al., 2017). Further *in vivo* studies as well as safety evaluations of these delivery systems will greatly promote the translational research of SFN from bench to bedside.

Secondly, the formulation is an important factor that affects the bioavailability and efficacy of SFN in humans. In most of the published clinical studies, broccoli sprout extracts or glucoraphanin-rich preparations were used instead of SFN. But administration of these formulations showed significant inter-individual variation of the bioavailability of SFN. Among which, the myrosinase-catalyzed conversion of glucoraphanin has demonstrated large differences in effect among individuals and is considered as an important factor that affects the bioavailability of SFN. Additionally, the precise dosages of SFN that subjects received from these formulations are difficult to quantify, and the efficacy of SFN in clinical trials may be influenced (Alumkal et al., 2015; Cipolla et al., 2015). The use of formulation containing stable and pure SFN is recommended for future studies due to its advantage of dose control and relative high bioavailability.

Thirdly, the selection of dose and dose schedule of SFN needs to be optimized in future studies. In previous studies, SFN or SFN-containing formulations were usually administered daily with a single dose. However, the half-life of SFN is very short due to its rapid metabolism in the human body. To date, there are few dose-response studies on SFN that have been reported and the range of its effective doses is unclear. Doses used in most animal studies have exceeded the highest dose of SFN used in humans. Thus, high quality dose-response research of SFN in the treatment of liver diseases are needed, which will provide valuable data for developing reasonable dose schedules of SFN in future clinical studies.

## 5 Conclusion

**FIGURE 4:**
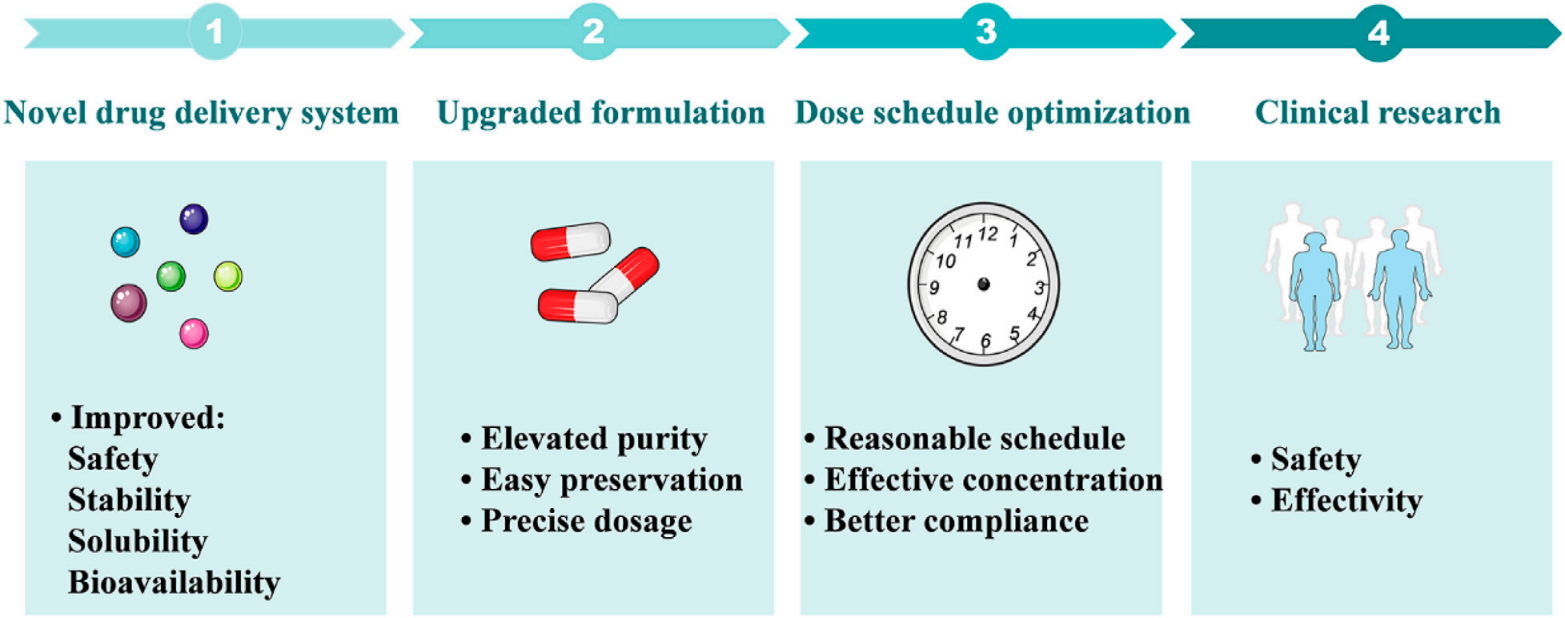
Future research perspectives of SFN in the treatment of liver diseases. 1: Novel drug delivery systems including nanoparticles, microencapsulation, and coated microparticles are expected to improve the solubility and stability of SFN, as well as its bioavailability and safety. 2: Advanced formulations will provide pure and stable SFN that is suitable for preservation and dose control. 3: Optimizing the dose schedule of SFN will provide reasonable dosing intervals, benefit the maintenance of effective blood concentration, and improve drug compliance. 4: Future clinical research focusing on the safety and efficacy of SFN in the treatment of liver diseases will facilitate its clinical transformation.

In this review, the therapeutic potential of SFN, a phytochemical derived from broccoli and other cruciferous vegetables, in the treatment of several liver diseases and related mechanisms were summarized and future research directions were discussed. In the past decades, both *in vitro* and *in vivo* studies have demonstrated significant benefits of SFN in the treatment of several liver diseases, including fatty liver disease, hepatic insulin resistance, liver injuries, and hepatocellular carcinoma. In terms of mechanism, Nrf2-mediated pathways play important roles in the therapeutic effects of SFN, such as Nrf2-mediated inhibition of lipogenesis and oxidative stress, anti-inflammation, and suppression of ER stress, Nrf2-mediated lipophagy, and mitochondrial function. Moreover, new regulatory pathways like FGF21-regulated enhancement of fatty acid metabolism, restoration of mitochondrial-ER stress interaction, and blocking of ceramide biosynthesis were found to be mediated by SFN. However, there is still a gap between the basic research and clinical application of SFN. More efficient delivery systems and precise dose schedules of SFN are expected to be developed in future studies, which would improve its solubility, stability, and bioavailability and reduce inter-individual variations in humans. And these future studies will greatly facilitate the translational research of SFN in the treatment of liver diseases and ultimately promote its clinical application (Figure 4).
